# Nonsurgical Rhinoplasty: Patient Satisfaction and Progression to Surgery

**DOI:** 10.1093/asjof/ojag043

**Published:** 2026-03-26

**Authors:** Kanad Ghosh, Lauren Greger, Alissa Zarder, Parit Patel

## Abstract

**Background:**

Nonsurgical rhinoplasty (NSR) may achieve a satisfactory aesthetic result. However, if NSR falls short of patient goals, patients may then elect to undergo surgical rhinoplasty. This latter cohort of patients has not been well studied in terms of satisfaction and motivation to pursue surgical rhinoplasty.

**Objectives:**

The objectives of this study are to determine when NSR may be most beneficial and why patients may choose to undergo surgical rhinoplasty following NSR.

**Methods:**

A retrospective review was conducted of patients undergoing NSR from 2019 to 2024 by a single surgeon. All patients received injection of hyaluronic acid filler. Demographic data, satisfaction scores using FACE-Q data, progression to surgical rhinoplasty, and complications following injection and rhinoplasty were recorded.

**Results:**

About one hundred and sixty-two patients were included in the study. About 90.7% were females, and average patient age was 30.5 ± 9.2 years. On average, patients had 1.7 ± 1.2 injections with an average of 0.38 + 0.08 cc volume per injection. Also, 8/162 (4.9%) of patients went on to surgical rhinoplasty with an average of 8.1 ± 5.8 months between last injection and surgery. The main motivations for proceeding to surgical rhinoplasty were patient readiness for more permanent results. FACE-Q surveys were completed by 36/162 (22.2%) of patients, which showed an average satisfaction of 79.8% + 15.7%.

**Conclusions:**

Nonsurgical rhinoplasty is a safe aesthetic procedure with low rate of progression to surgical rhinoplasty. In patients who elect to proceed to surgical rhinoplasty, the main motivation is readiness for permanent surgical outcomes.

**Level of Evidence: 3 (Therapeutic):**

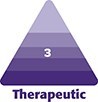

Surgical rhinoplasty is one of the most common and technically demanding procedures in Plastic Surgery. Techniques in improving surgical and aesthetic outcomes in rhinoplasty have been published for decades and continue to evolve. However, recent trends in the field have seen an increase in nonsurgical procedures in aesthetic surgery, namely increased use of injectables.^1^ Nonsurgical rhinoplasty (NSR), often referred to as “liquid rhinoplasty” is a procedure dating back to the early 2000s by which injectable filler is used to improve nasal aesthetics.^2^ It is especially useful for patients who require increased nasal projection, minor corrections of nasal asymmetry, or improved tip projection and rotation.^3,4^ It has been shown to improve patient satisfaction in specific indications without the need for surgery, which is more invasive, has significantly longer recovery time, and may lead to a higher rate of asymmetry, which requires revision surgery to correct.^5^

Though there have now been several studies examining injection protocols, complications from injections, and patient satisfaction after NSR, there are few studies that report on the progression of patients from NSR to surgical rhinoplasty. While studies by Ramos et al^6^ and others have detailed the technical aspects of surgical rhinoplasty after NSR, such as removal or reabsorption of filler prior to surgery for proper assessment of patient needs, few studies have reported on patient indications and reasoning for advancing from NSR to surgical rhinoplasty. In this study, we examine the clinical course of patients undergoing NSR and focus on patient-reported outcomes and progression to surgical rhinoplasty in select cohorts.

## METHODS

A retrospective chart review was conducted according to principles of the Declaration of Helsinki with signed patient consent for inclusion. All included patients presented for NSR by a single plastic surgeon from 2019 to 2024 via injection of hyaluronic acid (HA) filler (Juvéderm^®^ Ultra XC or Juvéderm^®^ Voluma [AbbVie, Chicago, IL]) into the external nose. Patients presenting for consultation of surgical rhinoplasty without prior NSR from the leading investigator or for consultation of any other procedure were excluded. Anatomic location of filler placement and amount of filler injected varied based on individual patient needs, but care was taken to inject midline avoiding lateral vascular structures, into the sub-SMAS plane, and with aspiration prior to injection. Patient demographics, medical history, and prior surgery including prior rhinoplasty were recorded. Number of injections and time between injections were evaluated, and time to surgical rhinoplasty in select patients was measured.

Patients were typically asked to fill out a FACE-Q survey to evaluate patient satisfaction following their first injection; some patients recorded their responses at this time, others elected to complete the survey after subsequent injections or elected not to complete the survey. No preinjection FACE-Q surveys were administered, and all FACE-Q data were completed prior to surgical rhinoplasty in patients who elected to have this procedure. Patient motivation to progress to surgical rhinoplasty was captured during patient interview and documented in individual clinic notes, which were reviewed during retrospective chart review.

Statistical analysis was conducted using SPSS (IBM) statistical software. Continuous variables were compared via independent *t*-test, and categorical variables compared via Chi-square test with a significant set to *P* < .05. Raw FACE-Q scores were taken and converted to a percent satisfaction for ease of interpretation.

### Injection Technique

Patients presenting for NSR were seen in the office setting. The nose was cleaned with an alcohol wipe, and Eutectic Mixture of Local Anesthetics (EMLA) cream was applied to the nose and allowed to sit for 15 minutes. After this, appropriate filler was chosen with consideration of the thickness of the patient's skin, ie, higher G′ fillers chosen for patients with thicker skin. Appropriate amount of filler was slowly injected into the nasal subunits according to patient goals, making sure to aspirate prior to injection and maintain a slow speed of injection. Filler was placed deep, close to the midline, and in a supraperiosteal or supraperichondreal plane. Patients were advised to apply ice to the nose postprocedure to reduce swelling and any redness, which was typically minimal.

## RESULTS

One hundred and sixty-two patients were included in our study. The average age was 30.5 ± 9.2 years (range 15-70), 147/162, 90.7% were women and 15/162, 9.3% were men. The average BMI was 22.0 ± 3.2. The majority of included patients were Caucasian (73/172, 45.1%), followed by Hispanic (39/162, 24.1%) and Asian (24/162, 14.8%; Table 1). On average, patients had 1.7 ± 1.2 injections with an average of 0.38 + 0.08 cc volume per injection and 175.4 ± 61.2 days in between injections (Table 2). Twenty-nine patients (17.9%) had a prior rhinoplasty on average 4.3 ± 5.7 years prior to their initial filler injection.

**Table 1.** Patient Demographics

	Nonsurgical Rhinoplasty (*n* = 162)
Age, years (average ± stdev)	30.5 ± 9.2
Male (*n*, %)	15, 9.3
Female (*n*, %)	147, 90.7
Ethnicity (*n*, %)	
Caucasian	73, 45.1
Hispanic	39, 24.1
Asian	24, 14.8
Black/African American	13, 8.0
Middle Eastern	13, 8.0

**Table 2.** Filler and Nonsurgical Rhinoplasty Details

	Nonsurgical Rhinoplasty (*n* = 162)
Filler type (*n*, %)	
Juvéderm^®^ Ultra XC	162 (98.2)
Juvéderm^®^ Voluma	3 (1.8)
Filler sessions (average ± stdev)	1.7 ± 1.2
Filler amount, cc (average ± stdev)	0.38 + 0.08
Time interval between filler, days (average ± stdev)	175.4 ± 61.2
Prior rhinoplasty (*n*, %)	29, 17.9
Rhinoplasty to filler, years (average ± stdev)	4.3 ± 5.7
Rhinoplasty after filler (*n*, %)	8, 4.9
Time interval between filler to rhinoplasty, months (average ± stdev)	8.1 ± 5.8
Filler after rhinoplasty (*n*, %)	1, 0.6

About 8/162 (4.9%) of patients went on to elect for surgical rhinoplasty following NSR with an average of 8.1 ± 5.8 months between last injection and surgery. In preparation for the surgical rhinoplasty, filler was first dissolved with hyaluronidase 3 months before the surgery. In most cases, residual filler was still found intraoperatively and was removed directly at the time of the surgery. Only one patient (0.6%) had any additional filler after a surgical rhinoplasty. Chart review of clinic visits prior to surgery revealed the main motivations for proceeding to surgical rhinoplasty were readiness of patients to undergo more permanent correction of nasal cosmesis after positive, but temporary, results with NSR. Comparison of demographics, number of NSR injections, and prior rhinoplasty in patients who underwent NSR and surgical rhinoplasty vs NSR alone revealed no significant differences (Table 3).

**Table 3.** Comparison of Patients With Nonsurgical Rhinoplasty (NSR) and Progression to Surgical Rhinoplasty Versus NSR Alone

	Progression to surgical rhinoplasty (*n* = 8)	NSR only (*n* = 154)	*P*-value
BMI (average ± stdev)	22.9 ± 2.4	21.9 ± 3.2	0.429
Age (average ± stdev)	29.4 ± 4.4	30.6 ± 9.4	0.721
Female (*n*, %)	8, 100	139, 90.3	0.354
Ethnicity (*n*, %)			0.812
Caucasian	3, 37.5	70, 45.5	
Middle Eastern	1, 12.5	12, 7.8	
Asian	2, 25.0	22, 14.3	
Black	1, 12.5	12, 7.8	
Hispanic	1, 12.5	38, 24.7	
Number of NSR injections (average ± stdev)	1.5 ± 0.5	1.8 ± 1.3	0.564
Prior rhinoplasty (*n*, %)	1, 12.5	28, 18.2	0.683

Review of complications showed one patient who experienced redness at the injection sites, which resolved with oral antibiotic treatment, and one patient with a history of 2 prior rhinoplasties, who developed early signs of vascular compromise after injection. The patient was immediately treated with hyaluronidase and topical nitroglycerine and subsequently had complete resolution of symptoms.

FACE-Q surveys were completed by 36/162 (22.2%) of patients, which showed an average satisfaction of 79.8% + 15.7%. Overall, patients had the highest satisfaction with nasal width (91.0%), and the least satisfaction with appearance of their nose from various angles (71.5%; Table 4). Comparison of FACE-Q responses and satisfaction revealed no significant differences between patients undergoing NSR and surgical rhinoplasty vs NSR alone (Table 5).

**Table 4.** FACE-Q Responses

	Nonsurgical rhinoplasty
FACE-Q respondents (*n*, %)	36/162, 22.2
Overall satisfaction (% ± stdev)	79.8 + 15.7
Satisfaction with (% ± stdev):	
Width	91.0 ± 16.0
Length	86.1 ± 17.4
Bridge	84.7 ± 21.8
Suits face	80.6 ± 17.0
Straight	80.6 ± 24.7
Size	72.2 ± 21.4
Shape	79.2 ± 22.8
Photos	76.4 ± 23.9
Tip	75.7 ± 22.7
Every angle	71.5 ± 21.7

**Table 5.** Comparison of FACE-Q Responses in Patients Undergoing Nonsurgical Rhinoplasty (NSR) With Progression to Rhinoplasty Versus NSR Alone

	Progression to surgical rhinoplasty (*n* = 3)	NSR only (*n* = 33)	*P*-value
FACE-Q scores (% satisfaction ± stdev)			
Width	83.3 ± 28.9	91.8 ± 14.9	0.395
Length	83.3 ± 28.9	86.3 ± 16.7	0.777
Bridge	91.8 ± 14.4	84.0 ± 22.4	0.572
Suits face	91.8 ± 14.4	84.0 ± 22.4	0.243
Straight	91.8 ± 14.4	79.5 ± 17.1	0.424
Size	75.0 ± 25.0	72.0 ± 21.4	0.818
Shape	91.8 ± 14.4	78.0 ± 23.2	0.327
Photos	91.8 ± 14.4	75 ± 24.2	0.252
Tip	91.8 ± 14.4	74.3 ± 23.0	0.209
Every angle	83.3 ± 28.9	70.5 ± 21.2	0.332

Of note, all FACE-Q responses were recorded prior to surgical rhinoplasty in the former group.

## DISCUSSION

Early reports of NSR date back to the 20th century, when physicians were using substances such as paraffin wax and silicone gel for nasal augmentation, which came with several complications such as ulcers and granulomas.^7^ In present times, HA (Juvéderm^®^, Restylane^®^ [Galderma, Zug, Switzerland]) and calcium hydroxyapatite (CaHa) (Radiesse^®^ [Merz Group, Frankfurt, Germany]) are the most used fillers for NSR. In most practices as in ours, HA is the choice of filler due to its reproducible results, longevity, and reversibility.^8,9^

However, NSR is not indicated in every patient and should not be seen as a “precursor” to surgical rhinoplasty—instead, NSR should be considered when it aligns with the goals and the anatomy of the patient. One aspect to consider in consultation is ethnicity. Harb et al,^8^ in a retrospective study of 487 African American patients, found the most common indications to be lack of bridge definition, excessive alar width, and bulbous tip. Though there are certainly differences within patients of the same ethnicity, average anatomical considerations associated with ethnicity and their contrast to a stereotypically Caucasian standard of beauty should be taken into account. This is reinforced by a recent systematic review by Atiyeh et al,^9^ which found that though there is often an emphasis on European beauty standards, patients typically prefer outcomes that align with their ethnic identity. The majority of patients in our cohort were Caucasian, Hispanic, and Asian; no significant differences were noted between ethnicities in our reported metrics nor between patients electing to undergo surgical rhinoplasty vs NSR alone. This may be due to the geographic location of our practice and surrounding demographics, and pooled data from multiple sites may be better suited to analyze demographic differences in progression to surgical rhinoplasty.

Beyond ethnicity, individual patient goals are important to recognize in considering whether NSR would be appropriate. Results of the FACE-Q data reported in our study show that NSR may be most helpful with concerns of nasal width, while it may not as useful in patients concerned about the size of their nose or the nasal tip. In studies of over 7000 aggregate patients, Harb et al found NSR to be successful in the correction of patients with nasal bridge collapse, dorsal hump correction, tip asymmetry, and tip under-projection.^8,10^ In a wider systematic review conducted by Santamaria Gadea et al,^3^ the authors found indications to be variable but mostly related to correction of deformities of the middle 1/3 of the nose; another systematic review by Mortada et al^11^ found similar indications, specifically correction of nasal hump deformities.

When counseling patients regarding results of NSR, it is useful to approximate how many sessions of injections patients typically need. In our patient cohort, the mean number of sessions 1.75, and 92/162 (56.8%) of patients only underwent one session. This is in concordance with a retrospective review conducted by Giammarioli et al,^12^ which found that 56% of their 101 patients required only one session with an 85% satisfaction rate. A retrospective review by Bertossi et al of 107 patients found a 30% volume contracture after 6 months and 50% decrease in volume after 12 months.^13,14^ In our patient cohort, most patients were initially not ready for the commitment of surgery for various reasons such as cost, recovery time, and concern for an invasive procedure. For the 8 patients in our cohort who proceeded to surgery, all were happy with the changes they saw with NSR and were finally ready to commit to a more permanent solution.

Though NSR is minimally invasive, there is still a serious risk of complication. The most common complication noted was erythema, swelling, and bruising.^10,15,16^ However, the most feared complication is ischemia and necrosis of the overlying skin, which can happen with failure to recognize vascular occlusion. This is most commonly due to intravascular injection and embolism of filler material, which also causes surrounding angiosomes to vasospasm.^17^ Signs and symptoms of vascular occlusion include immediate pain and blanching, a livedo pattern and delayed capillary refill in subsequent minutes, and skin breakdown over the course of days if left untreated. Aspirating before injection, slow injection technique, and small volume injections mitigate the risk of intravascular injection.^18^ If vascular occlusion is suspected, immediate injection of high dose hyaluronidase into the injection site as well as surrounding areas, along with massage and heat are recommended.

However, rates of skin necrosis remain low; in a retrospective review of 2275 patients, Rivkin et al^19^ found a 0.20% rate of ischemia and necrosis, similar to a 0.60% found in 492 patients by Jalali et al,^16^ and 0.27% found in a systematic review by Song et al.^15^ Complications in our cohort were minimal, consisting of one patient with signs of superficial infection treated with oral antibiotics and another patient with signs of acute vascular compromise at the injection site treated with hyaluronidase and topical nitroglycerine. Of note, the latter patient had a history of 2 prior rhinoplasties, which may compromise the vascularity of the nasal skin and increase the risk of vascular insult.

When evaluating patient-reported outcomes, there has been a reported high degree of satisfaction with NSR. Segreto et al^20^ compared preinjection and postinjection FACE-Q results in 70 patients and found significant increase in satisfaction in all surveyed questions. Bektas et al examined patient satisfaction in the setting of NSR alone, NSR after rhinoplasty, and rhinoplasty after NSR and found a high degree of satisfaction in all regards though there were no direct comparisons made in the study. Our data found a nearly 80% satisfaction with NSR after at least one session.^21^

Our data are limited due to its retrospective, single-center, single-surgeon design without a comparison group. Furthermore, we do not present an objective assessment of aesthetic outcome apart from patient satisfaction as reported by FACE-Q. It is further limited in not yet obtaining preinjection and postinjection data, or postsurgical FACE-Q data in patients who have undergone surgical rhinoplasty. Furthermore, only 22% of our included patient cohort completed the FACE-Q. The results of the survey are therefore limited by the low response rate and possible response bias, as patients with a more positive result may be more likely to respond to the FACE-Q. Also, given that only 3/8 patients who went on to surgical rhinoplasty completed the FACE-Q, comparisons made between this cohort and the nonsurgical cohort in terms of subjective satisfaction are under-powered. Lastly, patients may have been lost to follow up and elected to undergo surgical rhinoplasty with another provider, and thus not captured in our analysis.

## CONCLUSIONS

Our data show that NSR remains a safe cosmetic procedure with positive patient satisfaction among those surveyed and minimal complications. Only a small cohort of patients (4.9%) elected to undergo subsequent surgical rhinoplasty due to desire for more permanent results and readiness for surgical correction. Further studies with increased patient numbers done in a prospective nature may further elucidate the indications, safety, and patient satisfaction in NSR.
